# Early treatment of Class III malocclusion with facemask therapy

**DOI:** 10.1002/cre2.144

**Published:** 2018-11-18

**Authors:** Iván Menéndez‐Díaz, Juan Muriel, Juan L. Cobo, Covadonga Álvarez, Teresa Cobo

**Affiliations:** ^1^ Surgery and Medical‐Surgical Specialties Universidad de Oviedo Spain; ^2^ Orthodontics Division, Instituto Asturiano de Odontologia Universidad de Oviedo Spain; ^3^ Diagnostic Imaging Division, Instituto Asturiano de Odontologia Universidad de Oviedo Spain; ^4^ Maxillofacial Surgery Service Hospital Universitario Central de Asturias Spain

**Keywords:** facemask, maxillary hypoplasia, orthopedic treatment, skeletal effects, upper airways

## Abstract

The facemask is a widely used device in the treatment of Class III malocclusion and is intended to anteriorly displace the superior maxilla or stimulate its growth in that direction. The main goal of this study was to evaluate the effects of treatment using orthopedic maxillary expansion with facemask therapy in patients with Class III malocclusion. Sixty‐four patients, with a mean age of 8.14 ± 1.18 years at the start of treatment and a mean age of 9.78 ± 1.19 years at the end, were treated using orthopedic maxillary expansion and associated facemask therapy. The patients were evaluated using lateral head teleradiography before and after treatment, and the differences were analyzed. In addition, binary logistic regression was used as a model for predicting successful treatment. When comparing the changes achieved by treatment, statistically significant favorable changes were found at the skeletal level. Furthermore, an improvement in the airways at all levels was detected. Orthopedic maxillary expansion associated with facemask therapy has proven effective in treating early skeletal Class III malocclusion.

## 1 INTRODUCTION

Class III malocclusion is one of the most striking malocclusions and is hence usually identified early, because skeletal size differences already exist with respect to Class I occlusion at 4 to 5 years of age. In Class III malocclusion, the lower arch is more advanced in relation to the upper arch, and this condition worsens with age (Deguchi & Kageyama, 2014).

The orthopedic facemask and dentofacial procedure is widely employed for the treatment of skeletal Class III malocclusion. It seeks to anteriorly displace the maxilla or stimulate its growth in that direction. Furthermore, when used in combination with orthopedics, maxillary expansion not only favors the transversal development of the maxilla but also improves the sagittal effect (Foersch et al., 2015), because maxillary expansion significantly increases the palatine suture and other circumaxillary sutures (Ghoneima et al., 2011). Moreover, the forces generated by protracting the maxilla after orthopedic maxillary expansion are higher than when using a facemask alone and enhance the effects of the facemask (Gautam et al., 2009). With the disjunction associated with the facemask, a skeletal change and an increase in the length of the arcade are produced (Uzuner et al., 2017). In addition, at the level of the upper airways, orthopedic maxillary expansion leads to an increase in the size of the nasal cavity and the nasopharynx (Smith et al., 2012), and when the facemask is combined with orthopedic maxillary expansion, an increase in the volume of the airways is achieved, thus resulting in improved respiratory function (Auconi et al., 2015).

Skeletal Class III malocclusion should be treated early because the circumaxillary sutures are still not consolidated. This will ensure an enhanced orthopedic effect and greater long‐term stability, a reduction in the need for further complex treatments with a poorer prognosis in the patient's permanent dentition.

This study aimed to evaluate the effects of early treatment using orthopedic maxillary expansion combined with facemask therapy in patients with Class III malocclusion.

## 2 MATERIALS AND METHODS

Ninety growing subjects from the Instituto Asturiano de Odontología (IAO) were diagnosed with Class III malocclusion, after excluding those with a discrepancy between centric relation and maximum intercuspation. Consequently, a sample of 68 eligible patients was obtained for treatment using orthopedic maxillary expansion and facemask therapy. Thereafter, four patients were excluded because of the quality of the final radiographs. Thus, the final group consisted of 64 patients, 30 boys (46.87%) and 34 girls (53.12%), with a mean age of 8.14 ± 1.18 years at the start of treatment and 9.78 ± 1.19 years at the end of treatment. The control group consisted of the studies of 14 patients, eight men and six women, with a mean age of 8.21 ± 1.18 years of which lateral cranial radiography was available. The parents or legal representatives of the patients refused to be submitted to the proposed treatment, but they asked for a further diagnostic evaluation, with an included radiographic study, when the average age of the group was 10.14 ± 1.09.

In all cases, two lateral cephalometric skull radiographs were performed, one before treatment started (A) and another once it had ended (B). The orthopedic treatment consisted of a McNamara‐type acrylic palatal expander in combination with a facemask with 3/8″ 32 oz. elastics.

The total treatment time was 18 months and began with the placement of the palatal expander appliance, the design of which included vestibular hooks extending in a superior and anterior direction. Patients were instructed to activate the palatal expander with a pattern of a 1/4 turn every 3 days until the transversal problem was resolved. The time average of activation of the palatal expander was 3.47 ± 0.67 months, whereas the median decreased to 3 months. Patients were provided with an anterior‐protraction mask during or immediately after maxillary expansion and were advised to use the system for at least 14 hr a day. The average duration of treatment was 11.33 ± 1.77 months, whereas the median decreased to 10 months. The elastics joined the hooks in the palatal expander with the facemask bar generating orthopedic forces of 400 to 500 g per side.

All the radiographs analyzed were traced using Dolphin Imaging 11.7 Premium software (Dolphin Imaging & Management Solutions, USA). The cephalometric analysis generated 26 variables, of which nine were angular and 16 linear, and an index for each trace. Autodesk AutoCAD was used to calculate measures not incorporated in the aforementioned program, whereas the SolidWorks^®^ program, employing the plane view option, was used for the purposes of validation. The variables analyzed were cranial flexure, Co‐Point A, SNA, Point A to nasion perp, Co‐Gn, SNB, Pg to nasion perp, gonial angle, Wits appraisal, maxillo–mandibular difference, ANB, palatal plane, mandibular plane, ANS to Me, overjet, overbite, molar relationship, U1 to SN, L1 to palatal plane, VERT, PNS‐AD1, AD1‐Ba, PNS‐AD2, AD2‐H, and McNamara's upper and lower pharyngeal dimensions.

Differences in measurements before and after treatment were evaluated using Student's *t* test for paired samples, and the posttreatment change in biotype was assessed using Fisher's test. Depending on whether the assumption of normality was fulfilled or not, either Student's *t* test or the Wilcoxon test for independent samples was used to compare the treated group with the control group. The level of significance considered in the analyses was 0.05. The following binary logistic regression model was constructed to predict treatment success:

$$P\left(Y=1\;|\;\left(X_{1},X_{2},\ldots ,X_{r}\right)\right)=1/\left(1+\exp\left(-\left(\beta_{0}+\beta_{1}X_{1}+\cdots +\beta_{r}X_{r}\right)\right)\right).$$

Once the model was built and the coefficients of the equation obtained, goodness of fit was performed using the Hosmer–Lemeshow test to obtain pseudo *R* ^2^ coefficients and the area under the receiver operating characteristic curve. The statistical analysis was performed using the R software (R Development Core Team), version 3.2.8 (R: A language and environment for statistical computing [computer software manual], Vienna, Austria). To check the goodness of the regression model, the packages MKmisc, rms, and pscl were incorporated.

The studies and procedures were approved by the Research Ethics Committee of the IAO. This body analyzes problems and ethical values related to the work of IAO researchers in line with current legislation.

## 3 RESULTS

When comparing the changes before and after treatment using the paired‐samples *t* test, we found statistically significant changes in all cases except for the variables Pg to nasion perp, palatal plane inclination and mandibular plane angle (Table 1).

**Table 1:** Changes before and after treatment

Variables	Before treatment		After treatment		*P*‐value
	Mean	*SD*	Mean	*SD*	
Cranial flexure	120.45	5.18	121.9	5.26	0.002
Co‐Point A	72.18	3.91	76.91	3.87	<0.001
SNA	80.24	4.17	81.03	3.7	0.003
Point A‐nasion perp	0.09	2.87	1.07	2.8	<0.001
Co‐Gn	93.86	5.89	99.62	5.98	<0.001
SNB	78.41	3.84	77.67	3.36	0.003
Pg‐nasion perp	−2.57	5.38	−2.79	5.74	0.54
Gonial angle	126.4	7.37	125.17	6.58	0.02
Wits appraisal	−3.55	2.95	−1.38	2.63	<0.001
Maxillo–mandibular difference	21.33	4.59	22.77	3.81	<0.001
ANB	1.89	2.75	3.38	2.26	<0.001
Palatal plane	2.69	3.92	3.31	3.57	0.12
Mandibular plane	25	5.11	25.33	5.64	0.3
ANS‐Me	55.41	10.52	59.55	5.02	0.002
Overjet	−0.55	1.97	3.39	1.45	<0.001
Overbite	−0.03	2.08	1.52	1.72	<0.001
Molar relationship	−2.61	2.1	−0.45	2.07	<0.001
U1‐SN	98.34	6.9	102.35	6.06	<0.001
L1‐palatal plane	91.41	7.97	88.48	6.57	<0.001
PNS‐AD1	20.14	4.4	22.08	4.41	<0.001
AD1‐Ba	20.4	3.87	21.29	3.95	0.001
PNS‐AD2	14.26	4.04	17.10	4.45	<0.001
AD2‐H	13.34	3.12	14.03	3.65	0.01
Upper pharynx	6.72	2.42	8.64	2.27	<0.001
Lower pharynx	10.5	3.01	11.33	3	<0.001

*Note*. Values are expressed as the mean ± standard deviation.

When analyzing the patient's biotype, Fisher's test showed that differences occurred in the facial biotype after treatment (*P* < 0.001), although changes were not necessarily in the same direction: 10/31 subjects with a brachyfacial biotype became mesofacial after treatment; 11/13 subjects with a dolichofacial biotype maintained this status after treatment, though two acquired a mesofacial biotype; 14/20 subjects who were classified as mesofacial maintained this status after treatment, with two becoming brachyfacial and four becoming dolichofacial.

Differences at baseline between the treated group and the control group were analyzed, and the main results are summarized in Table 2. No significant differences were detected for any of the measures.

**Table 2:** Differences between the control group and the treated group at baseline

Variables	Control group	Treated group	*P*‐value
Cranial flexure	121.1 ± 5.37	120.45 ± 5.18	0.67
Co‐Point A	71 ± 4.39	72.18 ± 3.91	0.32
SNA	79.34 ± 3.08	80.24 ± 4.17	0.45
Point A‐nasion perp	−0.01 ± 3.47	0.09 ± 2.87	0.91
Co‐Gn	94.62 ± 6.54	93.86 ± 5.89	0.58
SNB	78.27 ± 3.61	78.41 ± 3.84	0.9
Pg‐nasion perp	−1.53 ± 7.57	−2.57 ± 5.38	0.55
Gonial angle	127.7 ± 6.17	126.4 ± 7.37	0.26
Wits appraisal	−4.21 ± 3.03	−3.55 ± 2.95	0.46
Maxillo–mandibular difference	23.36 ± 4.03	21.33 ± 4.59	0.1
ANB	1.06 ± 2.95	1.89 ± 2.75	0.32
Palatal plane	3.47 ± 4.65	2.69 ± 3.92	0.43
Mandibular plane	26.09 ± 5.88	25 ± 5.11	0.32
ANS‐Me	58.09 ± 3.04	55.41 ± 10.52	0.16
Overjet	−0.94 ± 2.69	−0.55 ± 1.97	0.54
Overbite	−1.03 ± 3.21	−0.03 ± 2.08	0.28
Molar relationship	−3.74 ± 2.84	−2.61 ± 2.1	0.17
U1‐SN	98.99 ± 8.79	98.34 ± 6.9	0.76
L1‐palatal plane	89.49 ± 11.53	91.41 ± 7.97	0.46
PNS‐AD1	19.46 ± 3.9	20.14 ± 4.4	0.59
AD1‐Ba	18.63 ± 2.77	20.4 ± 3.87	0.11
PNS‐AD2	13.36 ± 3.78	14.26 ± 4.04	0.31
AD2‐H	13.78 ± 3.25	13.34 ± 3.12	0.64
Upper pharynx	10.79 ± 2.46	10.5 ± 3.01	0.74
Lower pharynx	6.39 ± 2.13	6.72 ± 2.42	0.76

To determine what part of the changes was due to our treatment and what part to growth, we analyzed the differences between the treated and control groups. Statistically significant differences were seen in the variables Co‐Point A, Point A to nasion perp, SNB, Pg to nasion perp, Wits appraisal, maxillo–mandibular difference, ANB, overjet, overbite, molar relationship, and in all the airway variables studied (Table 3).

**Table 3:** Differences in changes produced in the control and treated groups

Variables	Control group	Treated group	*P*‐value
Cranial flexure	0.11 ± 1.97	1.45 ± 3.55	0.06
Co‐Point A	2.48 ± 1.28	4.74 ± 3.16	0.01
SNA	0 ± 1.5	0.8 ± 2.1	0.1
Point A‐nasion perp	−0.16 ± 0.94	0.98 ± 1.84	0.01
Co‐Gn	7.92 ± 4.19	5.76 ± 4.65	0.051
SNB	1.46 ± 2.24	−0.74 ± 1.91	<0.01
Pg‐nasion perp	2.75 ± 1.01	−0.22 ± 2.87	<0.01
Gonial angle	−1.86 ± 2.71	−1.23 ± 4.19	0.3
Wits appraisal	−1.86 ± 0.75	2.18 ± 2.46	<0.01
Maxillo–mandibular difference	3.26 ± 2.78	1.44 ± 3.15	0.02
ANB	−1.45 ± 1.8	1.49 ± 2.09	<0.01
Palatal plane	0.2 ± 0.46	0.62 ± 3.2	0.76
Mandibular plane	−0.29 ± 3.05	0.34 ± 2.62	0.5
ANS‐Me	4.87 ± 4.28	4.13 ± 10.07	0.13
Overjet	−1.04 ± 0.73	3.95 ± 1.96	<0.01
Overbite	−0.62 ± 0.78	1.55 ± 1.98	<0.01
Molar relationship	−2.15 ± 0.56	2.16 ± 2.17	<0.01
U1‐SN	6.59 ± 4.55	4.01 ± 6.61	0.17
L1‐palatal plane	−2.98 ± 7.84	−2.93 ± 5.89	0.47
PNS‐AD1	−0.34 ± 0.54	1.94 ± 2.56	<0.01
AD1‐Ba	−0.24 ± 0.84	0.89 ± 2.09	<0.01
PNS‐AD2	0.13 ± 1.03	2.83 ± 3.23	<0.01
AD2‐H	−0.25 ± 0.61	0.69 ± 2.11	<0.01
Upper pharynx	−0.44 ± 0.59	1.92 ± 1.91	<0.01
Lower pharynx	0.07 ± 0.58	0.83 ± 1.47	0.03

Treatment success was defined as any positive value for the difference of Wits appraisal from before to after treatment. The variables considered as predictors or independent variables in the regression model were the measurements in the initial cephalometric radiograph (A). Stepwise selection of variables was used, and the overjet and molar relationship variables were statistically significant. The exponential functions of the regression coefficients or odds ratio were 0.524 for overjet and 0.272 for the molar relationship, which indicates that the likelihood of success decreases with increasing overjet and the molar relationship at the start of treatment. The Hosmer–Lemeshow test was conducted to check the goodness of the constructed model, finding that the fit was satisfactory (*P*‐value = 0.99; Table 4). Furthermore, pseudo *R* ^2^ coefficients were obtained, and the area under the receiver operating characteristic curve was calculated, obtaining a value of 0.858; thus, the model satisfactorily distinguished between successful and unsuccessful cases (Table 5).

**Table 4:** Model coefficients along with their odds ratio, confidence intervals, and the significance of the Wald test

Variables	Coefficient	*P*‐value	OR	CI (95%)
Overjet	−0.646	0.008	0.524	(0.301, 0.810)
Molar ratio	−1.301	0.009	0.272	(0.085, 0.621)

*Note*. CI: confidence interval; OR: odds ratio.

**Table 5:** Pseudo‐*R* ^2^ coefficients

*R* ^2^ McFadden	*R* ^2^ Cox y Snell	*R* ^2^ Nagelkerke
0.3379	0.2782	0.4495

## 4 DISCUSSION

We studied the effectiveness of therapy using orthopedic maxillary expansion and facemask therapy for the early treatment of skeletal Class III malocclusion. Skeletal changes were observed over a period of 18 months, long‐term stability being sought by overcorrection of overjet, and the molar relationship. This is in good agreement with previous reports (Masucci et al., 2011; Anne Mandall et al., 2012). The main problem lies in the differential growth between the mandible and the maxilla (Marshall et al., 2011), which may be responsible for the recurrence of Class III malocclusion.

A limitation in our study was that the evaluation of the changes was carried out using only lateral cephalometric skull radiographs, as the Ethics Committee of the IAO prohibits conducting cone beam computed tomography on children for research purposes without specific justification.

Another limitation of our study that could incorporate bias is that based on the treatment of patients with a malocclusion, it is not possible from the ethical point of view to randomize patients to the treated group and the control group, which has a relevance if we consider that it is a malocclusion that involves aesthetic alterations when influencing the soft tissues, which can cause psychological and interpersonal problems (Becelli et al., 2002). For this reason, the control group was constituted taking advantage of studies of patients who refused to undergo the proposed treatment but again requested a further diagnostic evaluation with an included radiographic study.

In our study, we found that subjects who had a worse response to treatment, considering Wits appraisal as an indicator of treatment success, corresponded to those with a significant increase in mandibular size. This is because, although Class III maxillary malocclusion is usually the main culprit, there is a role played by the mandible in stability treatment. Severe maxillo–mandibular discrepancies, increased vertical dimension, and mandibular prognathism should be considered in the diagnosis and when planning treatment, as they are unfavorable factors for maintaining long‐term stability (Gu, 2010). Although these patients improve with treatment, they are candidates for orthognathic surgery in adulthood right from the outset. As a result, we should be cautious when it comes time to determine a long‐term prognosis and inform parents or guardians regarding the possibility of unfavorable development (Choi et al., 2017).

Several authors have reported an increase in the vertical dimension with the use of facemask therapy (Kwak et al., 2018) that might be reduced by protraction to a bone anchor instead of to a palatal expander with hooks (Koh & Chung, 2014; Maino et al., 2018). However, our results are in line with those of others who reported sagittal improvement regardless of the initial vertical skeletal pattern, who did not find any skeletal differences in the different biotypes (Pavoni et al., 2015). Our study did not demonstrate a significant increase in the vertical dimension, irrespective of the patient's initial biotype. Moreover, the increase in the intermaxillary vertical dimension during the period of protraction reported by other authors does not endure in long‐term studies (Baccetti et al., 2010).

There continues to be debate in regard to the airways. Some authors found no significant changes in the sagittal dimensions of the oropharynx or the nasopharynx (Baccetti et al., 2010), whereas others even claim that the expected increase in pharyngeal volume decreases (Pamporakis et al., 2014). Other authors reported an increase in volume in the nasopharynx (Lee et al., 2011; Kaygisiz et al., 2009; Sayinsu et al., 2006), the upper airways (Oktay & Ulukaya, 2008), the oropharynx, and the nasopharynx (Auconi et al., 2015). Our results showed statistically significant changes for all the values analyzed, showing an improvement in the airways at all levels.

There is no consensus as to the pattern of opening the expander screw. Several authors have concluded that patterns of a 1/4 turn per day and a 1/4 turn every 2 days have similar effects on dentofacial structures (Ramoglu & Sari, 2010). However, an activation pattern of a 1/4 turn every 3 days may be more stable than a faster pattern (Lagravere et al., 2005).

There is also a lack of consensus regarding the optimal force for anterior maxillary protraction, with reported values ranging from 180 to 800 g per side and duration ranging from 10 to 24 hr a day (Yepes et al., 2014). From our data analysis, we can state that an activation pattern of 1/4 turn every 3 days for palatal expansion (given that we are working with ages at which the palatal suture has not yet been consolidated), followed by the use of a facemask employing orthopedic forces of between 400 and 500 g per side and worn at least 14 h a day are sufficient to obtain skeletal effects for the correction of Class III malocclusion.

Moreover, there is very little evidence in the literature on the long‐term effects of changes produced by a facemask, which necessitates further studies that provide information on this point (Rongo et al., 2017; Woon & Thiruvenkatachari, 2017).

### WHY THIS PAPER IS IMPORTANT TO PEDIATRIC DENTISTS

- The results of this study demonstrate that the protocol of combining orthopedic maxillary expansion with facemask therapy produces significantly positive changes at both the skeletal level and the level of the airways.
- Although changes in the patient's biotype occur, this does not always become more vertical with treatment.
- Treatment success is conditioned by the initial situation and is limited by mandibular growth.
- Early treatment of skeletal Class III malocclusion is especially relevant for pediatric dentists, given that the results obtained in younger ages generally reduce the necessity for further complex treatments with a poorer prognosis in the permanent dentition.
